# The Challenge and Potential of Metagenomics in the Clinic

**DOI:** 10.3389/fimmu.2016.00029

**Published:** 2016-02-03

**Authors:** Heidi Mulcahy-O’Grady, Matthew L. Workentine

**Affiliations:** ^1^Infection Prevention and Control, Alberta Health Services, and Faculty of Medicine, Calgary, AB, Canada; ^2^Faculty of Veterinary Medicine, University of Calgary, Calgary, AB, Canada

**Keywords:** metagenomics, diagnositics, pathogen detection, *Clostridium difficile*, antibiotic resistance, bioinformatics, microbiome, clinical research

## Abstract

The bacteria, fungi, and viruses that live on and in us have a tremendous impact on our day-to-day health and are often linked to many diseases, including autoimmune disorders and infections. Diagnosing and treating these disorders relies on accurate identification and characterization of the microbial community. Current sequencing technologies allow the sequencing of the entire nucleic acid complement of a sample providing an accurate snapshot of the community members present in addition to the full genetic potential of that microbial community. There are a number of clinical applications that stand to benefit from these data sets, such as the rapid identification of pathogens present in a sample. Other applications include the identification of antibiotic-resistance genes, diagnosis and treatment of gastrointestinal disorders, and many other diseases associated with bacterial, viral, and fungal microbiomes. Metagenomics also allows the physician to probe more complex phenotypes such as microbial dysbiosis with intestinal disorders and disruptions of the skin microbiome that may be associated with skin disorders. Many of these disorders are not associated with a single pathogen but emerge as a result of complex ecological interactions within microbiota. Currently, we understand very little about these complex phenotypes, yet clearly they are important and in some cases, as with fecal microbiota transplants in *Clostridium difficile* infections, treating the microbiome of the patient is effective. Here, we give an overview of metagenomics and discuss a number of areas where metagenomics is applicable in the clinic, and progress being made in these areas. This includes (1) the identification of unknown pathogens, and those pathogens particularly hard to culture, (2) utilizing functional information and gene content to understand complex infections such as *Clostridium difficile*, and (3) predicting antimicrobial resistance of the community using genetic determinants of resistance identified from the sequencing data. All of these applications rely on sophisticated computational tools, and we also discuss the importance of skilled bioinformatic support for the implementation and use of metagenomics in the clinic.

## 1 Introduction

The complement of microorganisms that live on and within us, our microbiome, and its role in health and disease has become a central focus of current research. Research over the past few years has revealed how fundamentally intertwined we are with our microbial passengers. We have known for many years the connection to immune system development and gut health, but the impact of the microbiome on our health goes far beyond this (1). Metabolism (2), skin health (3–6), and even our mental health (7) have been shown to be influenced or to influence the microbiome. Many diseases, often complex and multi-factorial, such as allergy (8), asthma (9), inflammatory bowel disease (10), and even cardiovascular disease (11) and cancer (12, 13) have all been associated with alterations to the microbiome.

Shotgun sequencing of purified DNA, or metagenomics, is rapidly emerging as a powerful tool for both microbiology research and clinical applications due to the depth and breadth of information that can be acquired. The volume of DNA sequencing required to fully sequence a sample such as the human gut microbiome has traditionally made routine metagenomics unfeasible, particularly for diagnostics. However, the ever-increasing volumes and dropping prices are slowing bringing metagenomics into the clinician’s toolbox. One of the powerful aspects of metagenomics is the non-targeted nature of the sequencing. Once acquired, the DNA sequence can be queried for any number of interesting questions such as the presence of pathogens, metabolic pathways, antimicrobial resistance genes, and overall community composition. In this review, we discuss how this information is useful in a clinical context for both diagnostics and research with a particular emphasis on complex microbiome-associated phenotypes such as dysbiosis and *Clostridium difficile* infection. Furthermore, we make the case that if metagenomics is to be useful in the clinical context, skilled bioinformatic support will be essential, not just in the development of novel tools and algorithms but in the application of these tools and interpreting results. As sequencing technology continues to evolve rapidly, the most significant bottleneck for metagenomics (and other genomic analysis, for that matter) will not be sequencing, but the presence of skilled analysts to analyze the data and tease out the clinically relevant information is required.

## 2 What is Metagenomics?

Historically, microbial diversity has been primarily studied with culturing. Selective media and careful selection of culture conditions can recover tremendous range of organisms and is remarkably sensitive. In fact, culturing remains a valuable, albeit undervalued tool to study the microbiome (14–17). However, microbiologists noticed some time ago, a discrepancy in the number of organisms that could be counted under a microscope and the number that was able to be recovered in laboratory culture (18). This has led to the development of culture-independent techniques, based primarily on the analysis of single-subunit (SSU) rRNA genes. The reduced costs and ever-increasing read lengths of high-throughput sequencing have transformed this into an accessible and powerful technique. Sequencing of rRNA gene amplicons from hundreds of samples can be done in parallel, and these data are used to infer abundance and taxonomic profiles of the microbial species in a sample. Metagenomics, on the other hand, is the collective genetic material from all the genomes present in the sample (1) and provides a view into the functional potential of the population.

**Figure 1:**
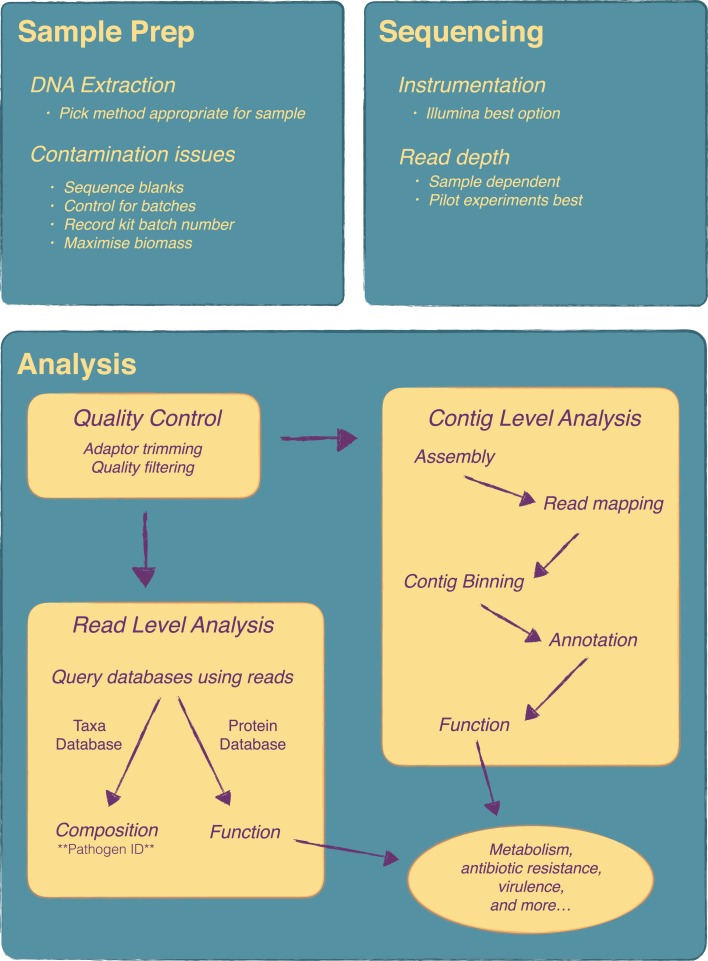
**Overview of a metagenomics experiment from sample preparation to analysis**. There are a number of key steps in a metagenomics experiment that require attention as they will influence the final results. Considerations on DNA extraction method and contamination issues are very important at the sample preparation stage. At the sequencing stage, the main consideration is read depth and pilot experiments are recommended for this. Analysis can be quite complex and will vary depending on the research or clinical question. Essentially, following quality control, analysis can be done at the contig level, i.e. following metagenome assembly, or directly at the read level.

The workflow for a metagenomics is fairly straightforward and is easily implemented in molecular biology laboratories. Several studies have been published recently that outline well many of the steps required when undertaking a microbiome study (19–23). A brief overview of the key steps for a metagenomics experiment is given here and is outlined in Figure 1.

### 2.1 DNA Extraction

The first step in a metagenomics experiment is the same as any other culture-independent method and that is to extract the DNA from the sample. Unfortunately, sample extraction gets little attention relative to other aspects of the workflow, yet can have a significant impact on outcome. A recent evaluation of extraction protocols from two major microbiome initiatives, the Human Microbiome Project (HMP) and metaHIT, revealed differences in the distribution of bacterial taxa as well as differences in gene composition based on which protocol the fecal samples were extracted with (24). Such differences are thought to arise in part from differential lysis due to cell wall composition (24) and will likely be even more pronounced for analysis that wish to evaluate a multi-kingdom microbiota that would include fungi. Therefore choice of extraction protocol is an important first step and will influence downstream results. Preliminary or pilot studies may choose to include a few different protocols to evaluate. For clinical applications, rigorous and systematic evaluation of different protocols for different tissues will no doubt be a key component of implementing a metagenomics workflow.

To improve the detection of target organisms, sample preparation can also include steps to enrich target sequences or remove un-wanted sequences. For example, detection of viruses can be improved by filtering out cellular material (25). Human DNA can be removed using laboratory methods to increase the amount of DNA coming from target sequences, although this often leaves small amounts of DNA remaining and requires newer kits to prepare the sequencing library (23).

### 2.2 Kit Contamination and Batch Effects

It is worth discussing in brief some recent work that has looked at the microbiome components that can be identified within the reagents and buffers of many commercial DNA extraction kits (26). The authors of this study sequenced the components of commonly used DNA extraction kits and demonstrated that contaminating DNA in these kit could significantly confound both 16S gene sequencing and metagenomics results, particularly low biomass samples. They went on to analyze a previously published study (27) demonstrating that the main finding in that study was completely confounded by which lot the extraction kit was from. Once the contaminating sequences were removed, the most significant feature of the data disappeared. Clearly batch effects are key issue for metagenomics studies, yet not often considered when designing these experiments. For example, a recent primer in cell (20) neglects to even mention batch effects, although the authors do include a section on potential contamination. Batch effects are widespread within high-throughput genomics experiments (28), and ameliorating and dealing with batch effects should be a key priority for any metagenomics experiment. Salter et al. (26) provide some recommendations (summarized in Figure 1), which mainly include sequencing reagent blanks as well as processing samples randomly if they need to be processed in multiple batches.

### 2.3 Sequencing Depth and Instrumentation

Although long-read sequencers from companies like PacBio and Oxford Nanopore have great potential for improving metagenomic datasets, particularly *de novo* assembly, these methods are currently too expensive to be practical. Hence Illumina’s short-read sequencers are currently the main choice for these types of experiments, and to acquire the read depth needed for good sample coverage, the higher output instruments such as the HiSeq and NextSeq are used. Previously, the main competitor for Illumina was a company called 454 (owned by Roche) whose sequencers could produce much longer reads (600–700 bp) as compared to Illumina’s 300 bp. However, in 2013, Roche announced it was closing 454 (https://www.genomeweb.com/sequencing/roche-shutting-down-454-sequencing-business) and phasing out all the sequencers. Currently, Illumina’s sequencers cannot be matched in terms of read quality and price-per-base and are really the only option for metagenomic studies. The ubiquity of Illumina’s sequencers across research laboratories and growing number of clinical laboratories makes them relatively accessible for both clinical and basic researchers.

The question then becomes one of read depth; how many reads are needed to answer a particular question. There has been some attempt to calculate the required sequencing depth based on the predicted taxonomic profile of the community of interest (29); however, currently there is no hard and fast rule for how much sequence to acquire and will depend on the desired outcome. For instance, if the sequencing is being done solely to identify an unknown pathogen, presumably this pathogen will be present in some numbers and may require less depth in order to detect it. The final choice of read depth will be highly dependent on experimental design and budget and is best determined empirically in pilot experiments.

### 2.4 Analysis

Generating sequencing data is done mostly with standard molecular biology protocols that are accessible to most research and clinical laboratories. However, analysis of metagenomic data is far from being standardized and is an area of active research and often the bottleneck for these types of studies. New methods are being published almost on a weekly basis, and a good analysis will invariably require a skilled analyst (see section at the end for more discussion on this point). Clinical applications of metagenomics are still limited by computational methods (see next section for a specific example); however, with good experimental design and the appropriate budget for analysis (often underestimated or neglected), the bioinformatics need not be prohibitory to successful application of metagenomics.

Figure 1 includes an overview of the main steps that may be included in a typical analysis. Very important is the initial quality control steps to ensure nothing went wrong with the sequencing. From there an analysis typically, depending on the experiment, can be divided up into different areas, although in most cases a variety of analysis will be done. Assigning taxonomy to the individual reads gives the taxonomic composition of the community, similar to what one would obtain from a 16S gene survey and is a key step for pathogen identification. There are quite a number of tools available to do this but few reach the speed and accuracy of Kraken (30) with MetaPhlAn as another common choice (31). To get functional composition, the reads are searched against a protein database such as KEGG. Again, a number of tools are available for this including web based tools such as MG-RAST (32) and MEGAN (33), which includes a graphical user interface. Some tools, such as HUMAnN (34), attempt to do metabolic reconstruction of the metagenomic data. As an alternative to read-level analysis, there is considerable work being done on metagenome assembly. A typical workflow might consist of a genome assembly with a specialized assembler such as Meta-IDBA (35) followed by binning contigs into groups with software like CONCOCT (36) and manual analysis and binning correction with a tool like Anvi’o (37). Contigs can then be annotated and subsequent functional analysis can be performed. Newer algorithms and tools are allowing for strain level analysis of metagenomic samples (38, 39), which holds much promise for infectious disease metagenomics as it allows the simultaneous identification of specific pathogenic strains and any corresponding antibiotic resistance and virulence genes these strains might carry.

## 3 Metagenomics for Diagnosis of Infectious Disease

Currently diagnosis of the vast majority of microbial diseases is carried out using traditional culture-based methods. In a clinical context, culture-based methods can fail to isolate disease-causing organism (40–42) and are time consuming and labor intensive (43). While not yet standard practice, utilizing a metagenomics approach in a clinical setting has the potential to identify and characterize bacterial and viral pathogens (44, 45). It is likely that reduced costing and increased sensitivity will endorse the use of culture-independent metagenomics approaches in clinical practice, particularly for new and emerging pathogens, which do not yet have standard diagnostic testing (44).

As discussed in the previous section, extracting useful information from these large datasets is non-trivial, often requiring multiple complex steps, which are dependent on the particulars of the data set being examined [e.g., see Ref. (44)]. Some inroads have been made in the development of tools for the rapid detection of pathogens in metagenomic datasets, which are designed to be fast and easy to use, important factors for integration into a clinical lab. PathSeq (46) utilizes a sequence subtraction method, where host DNA is first identified by aligning to a human reference database and removed leaving a much smaller dataset to search. This approach was used to identify a previously unknown pathogen as the causative agent in cord colitis syndrome (47). Clinical PathoScope (48) also uses the sequence subtraction method but claims to be much faster. Other computational strategies have been employed to deal with the large amount of host DNA; rapid identification of non-human sequences (RINS) (49) utilizes a custom reference database, which, while dramatically reduces run times requires some sort of hypothesis about the organism being identified. Parallel processing has also been used as a way to reduce computational times (50) while others have attempted to leverage improved alignment algorithms and increasingly available cloud computing resources (51). The later, known as sequence-based ultrarapid pathogen identification (SURPI) was recently used to diagnose a viral infection in a patient with encephalitis (52).

Given the rapid development of tools targeted for pathogen identification, it is feasible that this may be an area where metagenomics will play a key role in the clinical laboratory.

## 4 Metagenomics for Studying Dysbiosis and *Clostridium difficile* Infection

In addition to infection diagnostics, metagenomics has a great deal of potential for unraveling the microbial ecology of complicated disorders such as *Clostridium difficile* infection (CDI). The role of the microbiome in CDI is well studied, and CDI is considered as the prototypical example of a disease state, which occurs as a result of dysbiosis. Reduction of microbial diversity in the gut, most often as a result of antibiotic use (53, 54), results in reduced colonization resistance, promoting the overgrowth of *Clostridium difficile*. Using non-sequencing methods, it has been demonstrated that CDI patients had a decrease in the number of *Bacteroides*, *Prevotella*, and *Clostridia* groups IV and XIVa as well as higher levels of *Enterobacteriaceae* compared with healthy counterparts (55, 56). More recent sequencing studies, which provide a more in-depth analysis of the community structure, have demonstrated a less diverse gut microbiota in CDI patients relative to controls and CDI individuals demonstrated reductions in *Bacteroidaceae*, *Lachnospiraceae*, and *Ruminococcaceae* families (57–59).

In the clinic standard treatment of CDI is with metronidazole for mild disease or vancomycin for more severe disease (60). Both treatments are damaging to the normal microbiota, which contributes to the approximately 20% recurrence risk of disease (56). Therefore, new treatments for CDI must not only kill the pathogen but also simultaneously prevent destruction of the protective host microbial microflora. We are beginning to see studies looking at the microbiome sparing properties of new antibiotics (61) and anticancer drugs (62) in the literature, but it has been suggested that the going forward the Food and Drug administration could look at the host microbiota during clinical trial phase for all new drugs to determine their effect on the microbiome.

Remarkably, a lot of the information that has been gathered on the gut microbiota has come about as a result of studies on fecal microbiome transplantation (FMT). FMT is essentially transfer of the gut microbiota from the stool of healthy donor to sick patients. It has become widely used in the last decade due to its high success rate (up to 92–100% depending on the protocol used) (63, 64). In studies comparing recurrent CDI patients and pre- and post-FMT, it has been shown that intestinal microbiota changes from a low-diversity disease state dominated by *Streptococcaceae*, *Enterococcaceae*, and *Enterobacteriaceae* to a more diverse community, with significantly increased numbers of *Lachnospiraceae* and *Ruminococcaceae* (65, 66). However, although we know FMT works in the clinic we do not yet fully understand the specific mechanisms of why it works. Studies over the last two decades ago have demonstrated that bacterial mixtures of 6–33 different species can resolve recurrent *C. difficile* as effectively as whole fecal transplants (67–69). Additionally, recent germ free mouse studies have shown that a single *Lachnospiraceae* strain can suppress *C. difficile* infection in mice (70). Understanding the mechanism of what constitutes colonization resistance is made inherently more complex by the variability of the gut microbiome between people and the fact that identification of specific bacterial populations in the gut does not provide any concrete information regarding overall function.

The diversity of the human intestinal microbiome is a key to a number of biological processes that ensure the wellbeing of an individual. While certain bacterial species have been suggested as potential “keystone” species (71), it is likely that the functional state of the microbiome plays a more important role, rather then presence of a single species. Metagenomic sequencing offers a more comprehensive approach than marker gene approaches. Not only it can provide a complete view of the microbial community present but it also has the ability to resolve information about overall community function. Weingarden et al. (72) demonstrated that patients with recurrent CDI (rCDI) have high concentrations of primary bile acids and that FMT can restore the intestinal microbiota and the composition of fecal bile acids to that seen in non-CDI donor samples (72). Further evidence of a role for bile acids (BAs) was indicated by a recent study which identified *Clostridium scindens*, as an efficient inhibitor of CDI. This bacterium can convert primary BAs to secondary BAs thereby correcting the biosynthesis of secondary bile acids and inhibiting CDI.

Additional studies looking at the role of other metabolites in CDI have suggested butyrate deficiency in the colon increases growth and toxin production of *C. difficile* (73, 74). In turn, butyrate producers in the gut such as Lachnospiraceae and Ruminococcaceae are thought to have a protective role in preventing CDI. Paradoxically, butyrate has also been shown to be an activator of toxin synthesis in *C. difficile* (75). Furthermore, it was demonstrated using a gnotobiotic mouse model it that an abundance of commensally derived succinate allows *C. difficile* to expand efficiently and cause disease (76). In addition, genes involved in the conversion of succinate to butyrate were highly expressed under these conditions, suggesting a complex metabolic network is involved in pathogenesis. Finally, metagenomics will identify not only potentially beneficial bacteria and their functional role but also potential issues, such as antibiotic-resistance genes, or virulence genes; 16S RNA gene profiling is not sufficiently sensitive to differentiate between pathogenic and non-pathogenic or antibiotic-resistant and antibiotic-sensitive strains.

## 5 Understanding the Global Threat of Antimicrobial Resistance Using Metagenomics

Antimicrobial resistance is recognized as a growing global threat. Studying disruption of the human microbiome through use of antimicrobials is a topic of growing interest among healthcare professionals, because it could be a driving force behind the introduction and proliferation of antibiotic-resistant organisms (ARO) in health-care settings. In addition to CDI, microbial imbalance in the gut is the major predisposing factor for vancomycin-resistant enterococci (VRE) (77, 78) as well as other AROs including *Klebsiella* and *Escherchia coli* (53). If microbial imbalance is the major predisposing factor for infection with these organisms, then it stands to reason that a healthy microbiome is ones best defense against acquiring these organisms. Using a metagenomics approach, we can investigate the functional role of the host microbiome on the carriage and transmission of AROs; in patients who are asymptomatic carriers or actively infected with AROs, what is the state of their gut microbiome relative to non-carriers? Can we identify specific communities and functions that provide colonization resistance against some or all AROs?

In a diagnostic context, metagenomic data has tremendous potential for predicting antimicrobial sensitivity and resistance. Traditional methods of detecting antibiotic resistance suffer from the same problems as identifying pathogens given that these assays are performed on isolated organisms. However, the metagenome contains the collection of resistance determinants within the microbial community, known as the resistome (79), and can provide a comprehensive picture of the resistance “potential” of a community (80). Here, genetic determinants of resistance are identified in the data set and used to predict what resistance patterns will be. This works well for known mechanisms of resistance but would not be a useful for discovering novel methods or if the primary mechanism of resistance in a particular community is one that is unknown. The key advantage of a metagenomics approach is that all known determinants can be identified even ones that are not present in the disease causing pathogen, but may be transfered due to the highly mobile nature of many of these genes and pathways.

Although designed for whole genomes from single organisms, a great example of how this could work effectively is software called *Mykrobe predictor* (81), which uses de Bruijn graphs to identify a variety of different allele types, such as single-nucleotide polymorphisms (SNPs), indels and genes that are associated with antibiotic resistance. Using these data, a prediction is made on what antibiotics the organism will be resistant to. Although the authors demonstrate that it can be used to identify very minor alleles in a mixed infection it is at this stage, unlikely to work well in a complex infection environment such as the gut which would contain a fairly large number of antibiotic-resistance determinants from a variety of organisms (82, 83). Nonetheless, due to its speed, ease of use, and accuracy, this tool demonstrates how sequencing data can be used to make rapid predictions about antibiotic resistance very well and is a very promising step forward.

Identifying antibiotic-resistance genes in metagenomic datasets will depend strongly on the quality and completeness of resistance gene databases. Efforts such as the Comprehensive Antibiotic-Resistance Database (CARD) (84) and the Antibiotic-Resistance Gene Database (ARDB) (85) are extremely important for the appropriate interpretation of resistance levels of a sample based on gene content.

## 6 The Role of the Bioinformatician in Clinical Metagenomics

The semantics of definitions aside (86, 87), we put forward that if metagenomics is to be useful in a clinical context, it will require skilled bioinformatic analysts in addition to novel and efficient computational tools. Metagenomics can be immensely useful in clinical diagnostics as demonstrated by a study using a sequence-based metagenomics approach to investigate a shiga-toxigenic *Escherichia coli* (44) but such a study required a complex and non-standard analysis. Standard pipelines and tools with simple user interfaces can be setup and *Mykrobe predictor* (81) is a good example of this. Indeed as demonstrated by the number of pathogen identification tools intended to be user friendly there is considerable effort being put forth to remove complex analysis as a bottleneck. However, many datasets and analysis are not standard and require custom analysis. Furthermore, and perhaps more importantly, interpreting the output of analysis should never be done blindly, that is to say, a fundamental understanding of the tools and their limitations is paramount to acquiring accurate answers from metagenomic data in the clinic.

An advantage of metagenomics is that as new tools are created and new discoveries made the sequencing data can be utilized in ways previously unknown (Figure 1). In addition, new sequencing technologies, such as single-molecule sequencing, which produces very long reads, are emerging and established tools may or may not work with these new types of data. Having skilled bioinformaticians will become even more important as clinical labs and research become more and more dependent on sequencing data.

## 7 Conclusion

Metagenomics holds much promise for microbial diagnostics and research and there are several exiting proof-of-concept studies demonstrating the power of this approach for the clinical laboratory. Decreasing costs and increasing throughput will likely remove sequencing as a bottleneck leaving computational power, effective tools, and timely analysis as key issues that will need to be addressed to see to the full potential of metagenomic sequencing in the clinic.

## Author Contributions

All authors listed, have made substantial, direct and intellectual contribution to the work, and approved it for publication.

## Conflict of Interest Statement

The authors declare that the research was conducted in the absence of any commercial or financial relationships that could be construed as a potential conflict of interest.

## References

[1] Cho I Blaser MJ. The human microbiome: at the interface of health and disease. Nat Rev Genet (2012) 13:260–70. 10.1038/nrg3182 22411464 PMC3418802

[2] Tremaroli V Bäckhed F. Functional interactions between the gut microbiota and host metabolism. Nature (2012) 489:242–9. 10.1038/nature11552 22972297

[3] Weyrich LS Dixit S Farrer AG Cooper AJ. The skin microbiome: associations between altered microbial communities and disease. Australas J Dermatol (2015) 56:268–74. 10.1111/ajd.12253 25715969

[4] SanMiguel A Grice EA. Interactions between host factors and the skin microbiome. Cell Mol Life Sci (2015) 72:1499–515. 10.1007/s00018-014-1812-z 25548803 PMC4376244

[5] Oh J Byrd AL Deming C Conlan S NISC Comparative Sequencing Program, Kong HH Biogeography and individuality shape function in the human skin metagenome. Nature (2014) 514:59–64. 10.1038/nature13786 25279917 PMC4185404

[6] Schloss PD Microbiology: an integrated view of the skin microbiome. Nature (2014) 514:44–5. 10.1038/514044a 25279916

[7] Cryan JF Dinan TG. Mind-altering microorganisms: the impact of the gut microbiota on brain and behaviour. Nat Rev Neurosci (2012) 13:701–12. 10.1038/nrn3346 22968153

[8] Trompette A Gollwitzer ES Yadava K Sichelstiel AK Sprenger N Ngom-Bru C Gut microbiota metabolism of dietary fiber influences allergic airway disease and hematopoiesis. Nat Med (2014) 20:159–66. 10.1038/nm.3444 24390308

[9] Huang YJ Boushey HA. The microbiome and asthma. Ann Am Thorac Soc (2014) 11:S48–51. 10.1513/AnnalsATS.201306-187MG 24437406 PMC3972976

[10] Cammarota G Ianiro G Cianci R Bibbò S Gasbarrini A Currò D. The involvement of gut microbiota in inflammatory bowel disease pathogenesis: potential for therapy. Pharmacol Ther (2015) 149:191–212. 10.1016/j.pharmthera.2014.12.006 25561343

[11] Ordovas JM Mooser V. Metagenomics: the role of the microbiome in cardiovascular diseases. Curr Opin Lipidol (2006) 17:157–61. 10.1097/01.mol.0000217897.75068.ba 16531752

[12] Garrett WS Cancer and the microbiota. Science (2015) 348:80–6. 10.1126/science.aaa4972 25838377 PMC5535753

[13] Bultman SJ. Emerging roles of the microbiome in cancer. Carcinogenesis (2014) 35:249–55. 10.1093/carcin/bgt392 24302613 PMC3908754

[14] Goodman AL Kallstrom G Faith JJ Reyes A Moore A Dantas G Extensive personal human gut microbiota culture collections characterized and manipulated in gnotobiotic mice. Proc Natl Acad Sci USA (2011) 108:6252–7. 10.1073/pnas.1102938108 21436049 PMC3076821

[15] Stewart EJ. Growing unculturable bacteria. J Bacteriol (2012) 194:4151–60. 10.1128/JB.00345-12 22661685 PMC3416243

[16] Sibley CD Grinwis ME Field TR Eshaghurshan CS Faria MM Dowd SE Culture enriched molecular profiling of the cystic fibrosis airway microbiome. PLoS One (2011) 6:e22702. 10.1371/journal.pone.0022702 21829484 PMC3145661

[17] Lagier JC Armougom F Million M Hugon P Pagnier I Robert C Microbial culturomics: paradigm shift in the human gut microbiome study. Clin Microbiol Infect (2012) 18:1185–93. 10.1111/1469-0691.12023 23033984

[18] Staley JT Konopka A Measurement of in situ activities of nonphotosynthetic microorganisms in aquatic and terrestrial habitats. Annu Rev Microbiol (1985) 39:321–46. 10.1146/annurev.mi.39.100185.001541 3904603

[19] Vo ATE Jedlicka JA. Protocols for metagenomic DNA extraction and Illumina amplicon library preparation for faecal and swab samples. Mol Ecol Resour (2014) 14:1183–97. 10.1111/1755-0998.12269 24774752

[20] Goodrich JK Di Rienzi SC Poole AC Koren O Walters WA Caporaso JG Conducting a microbiome study. Cell (2014) 158:250–62. 10.1016/j.cell.2014.06.037 25036628 PMC5074386

[21] Knight R Jansson J Field D Fierer N Desai N Fuhrman JA Unlocking the potential of metagenomics through replicated experimental design. Nat Biotechnol (2012) 30:513–20. 10.1038/nbt.2235 22678395 PMC4902277

[22] Thomas T Gilbert J Meyer F. Metagenomics – a guide from sampling to data analysis. Microb Inform Exp (2012) 2:3. 10.1186/2042-5783-2-3 22587947 PMC3351745

[23] Miller RR Montoya V Gardy JL Patrick DM Tang P. Metagenomics for pathogen detection in public health. Genome Med (2013) 5:81. 10.1186/gm485 24050114 PMC3978900

[24] Wesolowska-Andersen A Bahl MI Carvalho V Kristiansen K Sicheritz-Ponten T Gupta R Choice of bacterial DNA extraction method from fecal material influences community structure as evaluated by metagenomic analysis. Microbiome (2014) 2:1. 10.1186/2049-2618-2-19 24949196 PMC4063427

[25] Thurber RV Haynes M Breitbart M Wegley L Rohwer F. Laboratory procedures to generate viral metagenomes. Nat Protoc (2009) 4:470–83. 10.1038/nprot.2009.10 19300441

[26] Salter SJ Cox MJ Turek EM Calus ST Cookson WO Moffatt MF Reagent and laboratory contamination can critically impact sequence-based microbiome analyses. BMC Biol (2014) 12:1. 10.1186/s12915-014-0087-z 25387460 PMC4228153

[27] Turner P Turner C Jankhot A Helen N Lee SJ Day NP A longitudinal study of *Streptococcus pneumoniae* carriage in a cohort of infants and their mothers on the Thailand-Myanmar border. PLoS One (2012) 7:e38271 10.1371/journal.pone.0038271 22693610 PMC3365031

[28] Leek JT Scharpf RB Bravo HC Simcha D Langmead B Johnson WE Tackling the widespread and critical impact of batch effects in high-throughput data. Nat Rev Genet (2010) 11:733–9. 10.1038/nrg2825 20838408 PMC3880143

[29] Ni J Yan Q Yu Y. How much metagenomic sequencing is enough to achieve a given goal? Sci Rep (2013) 3:1–7. 10.1038/srep01968 23752679 PMC3678137

[30] Wood DE Salzberg SL. Kraken: ultrafast metagenomic sequence classification using exact alignments. Genome Biol (2014) 15:R46. 10.1186/gb-2014-15-3-r46 24580807 PMC4053813

[31] Segata N Waldron L Ballarini A Narasimhan V Jousson O Huttenhower C. Metagenomic microbial community profiling using unique clade-specific marker genes. Nat Methods (2012) 9:811–4. 10.1038/nmeth.2066 22688413 PMC3443552

[32] Meyer F Paarmann D D’Souza M Olson R Glass EM Kubal M The metagenomics RAST server a public resource for the automatic phylogenetic and functional analysis of metagenomes. BMC Bioinformatics (2008) 9:1 10.1186/1471-2105-9-386 18803844 PMC2563014

[33] Huson DH Mitra S Ruscheweyh H-J Weber N Schuster SC. Integrative analysis of environmental sequences using MEGAN4. Genome Res (2011) 21:1552–60. 10.1101/gr.120618.111 21690186 PMC3166839

[34] Abubucker S Segata N Goll J Schubert AM Izard J Cantarel BL Metabolic reconstruction for metagenomic data and its application to the human microbiome. PLoS Comput Biol (2012) 8:e1002358. 10.1371/journal.pcbi.1002358 22719234 PMC3374609

[35] Peng Y Leung HCM Yiu SM Chin FYL. Meta-IDBA: a de novo assembler for metagenomic data. Bioinformatics (2011) 27:i94–101. 10.1093/bioinformatics/btr216 21685107 PMC3117360

[36] Alneberg J Bjarnason BS de Bruijn I Schirmer M Quick J Ijaz UZ Binning metagenomic contigs by coverage and composition. Nat Methods (2014) 11:1144–6. 10.1038/nmeth.3103 25218180

[37] Eren AM Esen ÖC Quince C Vineis JH Morrison HG Sogin ML Anvio: an advanced analysis and visualization platform for omics data. PeerJ (2015) 3:e1319 10.7717/peerj.1319 26500826 PMC4614810

[38] Luo C Knight R Siljander H Knip M Xavier RJ Gevers D. Constrains identifies microbial strains in metagenomic datasets. Nat Biotechnol (2015) 33:1045–52. 10.1038/nbt.3319 26344404 PMC4676274

[39] Nayfach S Pollard KS Population genetic analyses of metagenomes reveal extensive strain-level variation in prevalent human-associated bacteria. bioRxiv (2015):031757 10.1101/031757

[40] Dreier J Vollmer T Freytag CC Bäumer D Körfer R Kleesiek K. Culture-negative infectious endocarditis caused by *Bartonella* spp.: 2 case reports and a review of the literature. Diagn Microbiol Infect Dis (2008) 61:476–83. 10.1016/j.diagmicrobio.2008.03.008 18455348

[41] Richardson DC Burrows LL Korithoski B Salit IE Butany J David TE *Tropheryma whippelii* as a cause of afebrile culture-negative endocarditis: the evolving spectrum of Whipple’s disease. J Infect (2003) 47:170–3. 10.1016/S0163-4453(03)00015-X 12860154

[42] Nakamura S Maeda N Miron IM Yoh M Izutsu K Kataoka C Metagenomic diagnosis of bacterial infections. Emerg Infect Dis (2008) 14:1784–6. 10.3201/eid1411.080589 18976571 PMC2630750

[43] Pallen MJ Loman NJ Penn CW. High-throughput sequencing and clinical microbiology: progress, opportunities and challenges. Curr Opin Microbiol (2010) 13:625–31. 10.1016/j.mib.2010.08.003 20843733

[44] Loman NJ Constantinidou C Christner M Rohde H Chan JZ-M Quick J A culture-independent sequence-based metagenomics approach to the investigation of an outbreak of shiga-toxigenic *Escherichia coli* O104:H4. JAMA (2013) 309:1502–10. 10.1001/jama.2013.3231 23571589

[45] Mokili JL Rohwer F Dutilh BE. Metagenomics and future perspectives in virus discovery. Curr Opin Virol (2012) 2:63–77. 10.1016/j.coviro.2011.12.004 22440968 PMC7102772

[46] Kostic AD Ojesina AI Pedamallu CS Jung J Verhaak RGW Getz G PathSeq: software to identify or discover microbes by deep sequencing of human tissue. Nat Biotechnol (2011) 29:393–6. 10.1038/nbt.1868 21552235 PMC3523678

[47] Bhatt AS Freeman SS Herrera AF Pedamallu CS Gevers D Duke F Sequence-based discovery of *Bradyrhizobium entericain* cord colitis syndrome. N Engl J Med (2013) 369:517–28. 10.1056/NEJMoa1211115 23924002 PMC3889161

[48] Byrd AL Perez-Rogers JF Manimaran S Castro-Nallar E Toma I McCaffrey T Clinical pathoscope: rapid alignment and filtration for accurate pathogen identification in clinical samples using unassembled sequencing data. BMC Bioinformatics (2014) 15:262. 10.1186/1471-2105-15-262 25091138 PMC4131054

[49] Bhaduri A Qu K Lee CS Ungewickell A Khavari PA. Rapid identification of non-human sequences in high-throughput sequencing datasets. Bioinformatics (2012) 28:1174–5. 10.1093/bioinformatics/bts100 22377895 PMC3324519

[50] Naeem R Rashid M Pain A READSCAN: a fast and scalable pathogen discovery program with accurate genome relative abundance estimation. Bioinformatics (2013) 29:391–2. 10.1093/bioinformatics/bts684 23193222 PMC3562070

[51] Naccache SN Federman S Veeraraghavan N Zaharia M Lee D Samayoa E A cloud-compatible bioinformatics pipeline for ultrarapid pathogen identification from next-generation sequencing of clinical samples. Genome Res (2014) 24:1180–92. 10.1101/gr.171934.113 24899342 PMC4079973

[52] Naccache SN Peggs KS Mattes FM Phadke R Garson JA Grant P Diagnosis of neuroinvasive astrovirus infection in an immunocompromised adult with encephalitis by unbiased next-generation sequencing. Clin Infect Dis (2015) 60:919–23. 10.1093/cid/ciu912 25572898 PMC4345816

[53] De La Cochetière MF Durand T Lalande V Petit JC Potel G Beaugerie L. Effect of antibiotic therapy on human fecal microbiota and the relation to the development of *Clostridium difficile*. Microb Ecol (2008) 56:395–402. 10.1007/s00248-007-9356-5 18209965

[54] Deakin LJ Clare S Fagan RP Dawson LF Pickard DJ West MR The *Clostridium difficile* spo0A Gene is a persistence and transmission factor. Infect Immun (2012) 80:2704–11. 10.1128/IAI.00147-12 22615253 PMC3434595

[55] Hopkins MJ Sharp R Macfarlane GT. Age and disease related changes in intestinal bacterial populations assessed by cell culture, 16S rRNA abundance, and community cellular fatty acid profiles. Gut (2001) 48:198–205. 10.1136/gut.48.2.198 11156640 PMC1728209

[56] Louie TJ Byrne B Emery J Ward L Krulicki W Nguyen D Differences of the fecal microflora with *Clostridium difficile* therapies. Clin Infect Dis (2015) 60(Suppl 2):S91–7. 10.1093/cid/civ252 25922407

[57] Antharam VC Li EC Ishmael A Sharma A Mai V Rand KH Intestinal dysbiosis and depletion of butyrogenic bacteria in *Clostridium difficile* infection and nosocomial diarrhea. J Clin Microbiol (2013) 51:2884–92. 10.1128/JCM.00845-13 23804381 PMC3754663

[58] Schubert AM Rogers MAM Ring C Mogle J Petrosino JP Young VB Microbiome data distinguish patients with *Clostridium difficile* infection and non-*C. difficile*-associated diarrhea from healthy controls. MBio (2014) 5:e1021–1014. 10.1128/mBio.01021-14 24803517 PMC4010826

[59] Rea MC O’Sullivan O Shanahan F O’Toole PW Stanton C Ross RP *Clostridium difficile* carriage in elderly subjects and associated changes in the intestinal microbiota. J Clin Microbiol (2012) 50:867–75. 10.1128/JCM.05176-11 22162545 PMC3295116

[60] Johnson S Louie TJ Gerding DN Cornely OA Chasan-Taber S Fitts D Vancomycin, metronidazole, or tolevamer for *Clostridium difficile* infection: results from two multinational, randomized, controlled trials. Clin Infect Dis (2014) 59:345–54. 10.1093/cid/ciu313 24799326

[61] Louie TJ Cannon K Byrne B Emery J Ward L Eyben M Fidaxomicin preserves the intestinal microbiome during and after treatment of *Clostridium difficile* infection (CDI) and reduces both toxin reexpression and recurrence of CDI. Clin Infect Dis (2012) 55(Suppl 2):S132–42. 10.1093/cid/cis338 22752862 PMC3388020

[62] Viaud S Saccheri F Mignot G Yamazaki T Daillère R Hannani D The intestinal microbiota modulates the anticancer immune effects of cyclophosphamide. Science (2013) 342:971–6. 10.1126/science.1240537 24264990 PMC4048947

[63] Gough E Shaikh H Manges AR. Systematic review of intestinal microbiota transplantation (fecal bacteriotherapy) for recurrent *Clostridium difficile* infection. Clin Infect Dis (2011) 53:994–1002. 10.1093/cid/cir632 22002980

[64] Louie TJ Cannon K O’Grady H Wu K Ward L Fecal microbiome transplantation (FMT) via oral fecal microbial capsules for recurrent *Clostridium difficile* infection (rCDI). Oral Abstract Session: New Considerations in C. difficile Prevention and Treatment. San Diego, CA: ID week 2013 (2013). Available from: https://idsa.confex.com/idsa/2013/webprogram/Paper41627.html

[65] Song Y Garg S Girotra M Maddox C von Rosenvinge EC Dutta A Microbiota dynamics in patients treated with fecal microbiota transplantation for recurrent *Clostridium difficile* infection. PLoS One (2013) 8:e81330. 10.1371/journal.pone.0081330 24303043 PMC3841263

[66] Fuentes S van Nood E Tims S Heikamp-de Jong I ter Braak CJ Keller JJ Reset of a critically disturbed microbial ecosystem: faecal transplant in recurrent *Clostridium difficile* infection. ISME J (2014) 8:1621–33. 10.1038/ismej.2014.13 24577353 PMC4817604

[67] Lawley TD Clare S Walker AW Stares MD Connor TR Raisen C Targeted restoration of the intestinal microbiota with a simple, defined bacteriotherapy resolves relapsing *Clostridium difficile* disease in mice. PLoS Pathog (2012) 8:e1002995. 10.1371/journal.ppat.1002995 23133377 PMC3486913

[68] Tvede M Rask-Madsen J. Bacteriotherapy for chronic relapsing *Clostridium difficile* diarrhoea in six patients. Lancet (1989) 1:1156–60. 10.1016/S0140-6736(89)92749-9 2566734

[69] Petrof EO Gloor GB Vanner SJ Weese SJ Carter D Daigneault MC Stool substitute transplant therapy for the eradication of *Clostridium dif ficile* infection: ‘RePOOPulating’ the gut. Microbiome (2013) 1:3 10.1186/2049-2618-1-3 24467987 PMC3869191

[70] Reeves AE Koenigsknecht MJ Bergin IL Young VB. Suppression of *Clostridium difficile* in the gastrointestinal tracts of germfree mice inoculated with a murine isolate from the family lachnospiraceae. Infect Immun (2012) 80:3786–94. 10.1128/IAI.00647-12 22890996 PMC3486043

[71] Power ME Tilman D Estes JA Menge BA Bond WJ Challenges in the quest for keystones. Bioscience (1996) 46(8):609–20. 10.2307/1312990

[72] Weingarden AR Chen C Bobr A Yao D Lu Y Nelson VM Microbiota transplantation restores normal fecal bile acid composition in recurrent *Clostridium difficile* infection. Am J Physiol Gastrointest Liver Physiol (2014) 306:G310–9. 10.1152/ajpgi.00282.2013 24284963 PMC3920123

[73] O’Keefe SJD. Tube feeding, the microbiota, and *Clostridium difficile* infection. World J Gastroenterol (2010) 16:139–42. 10.3748/wjg.v16.i2.139 20066732 PMC2806551

[74] May T Mackie RI Fahey GC Cremin JC Garleb KA. Effect of fiber source on short-chain fatty acid production and on the growth and toxin production by *Clostridium difficile*. Scand J Gastroenterol (1994) 29:916–22. 10.3109/00365529409094863 7839098

[75] Bouillaut L Dubois T Sonenshein AL Dupuy B. Integration of metabolism and virulence in *Clostridium difficile*. Res Microbiol (2015) 166:375–83. 10.1016/j.resmic.2014.10.002 25445566 PMC4398617

[76] Ferreyra JA Wu KJ Hryckowian AJ Bouley DM Weimer BC Sonnenburg JL. Gut microbiota-produced succinate promotes *C. difficile* infection after antibiotic treatment or motility disturbance. Cell Host Microbe (2014) 16:770–7. 10.1016/j.chom.2014.11.003 25498344 PMC4859344

[77] Bhalla A Pultz NJ Ray AJ Hoyen CK Eckstein EC Donskey CJ. Antianaerobic antibiotic therapy promotes overgrowth of antibiotic-resistant, gram-negative bacilli and vancomycin-resistant *Enterococci* in the stool of colonized patients. Infect Control Hosp Epidemiol (2003) 24:644–9. 10.1086/502267 14510245

[78] Ubeda C Taur Y Jenq RR Equinda MJ Son T Samstein M Vancomycin-resistant *Enterococcus* domination of intestinal microbiota is enabled by antibiotic treatment in mice and precedes bloodstream invasion in humans. J Clin Invest (2010) 120:4332–41. 10.1172/JCI43918 21099116 PMC2993598

[79] Wright GD. The antibiotic resistome: the nexus of chemical and genetic diversity. Nat Rev Microbiol (2007) 5:175–86. 10.1038/nrmicro1614 17277795

[80] Schmieder R Edwards R. Insights into antibiotic resistance through metagenomic approaches. Future Microbiol (2012) 7:73–89. 10.2217/fmb.11.135 22191448

[81] Bradley P Gordon NC Walker TM Dunn L Heys S Huang B Rapid antibiotic resistance predictions from genome sequence data for *S*. *aureus* and *M*. *tuberculosis*. bioRxiv (2015):018564 10.1101/018564 PMC4703848 26686880

[82] Hu Y Yang X Qin J Lu N Cheng G Wu N Metagenome-wide analysis of antibiotic resistance genes in a large cohort of human gut microbiota. Nat Commun (2013) 4:2151. 10.1038/ncomms3151 23877117

[83] Forslund K Sunagawa S Coelho LP Bork P. Metagenomic insights into the human gut resistome and the forces that shape it. Bioessays (2014) 36:316–29. 10.1002/bies.201300143 24474281

[84] McArthur AG Waglechner N Nizam F Yan A Azad MA Baylay AJ The comprehensive antibiotic resistance database. Antimicrob Agents Chemother (2013) 57:3348–57. 10.1128/AAC.00419-13 23650175 PMC3697360

[85] Liu B Pop M ARDB–Antibiotic resistance genes database. Nucleic Acids Res (2009) 37:D443–7. 10.1093/nar/gkn656 18832362 PMC2686595

[86] Vincent AT Charette SJ Who qualifies to be a bioinformatician? Front Genet (2015) 6:164 10.3389/fgene.2015.00164 25964799 PMC4408859

[87] Smith DR Broadening the definition of a bioinformatician. Front Genet (2015) 6:258 10.3389/fgene.2015.00258 26300909 PMC4523842

